# Osteopontin Rejuvenates Senescent Adipose-Derived Stem Cells and Restores their Bone Tissue Regenerative Function

**DOI:** 10.1007/s12015-024-10707-5

**Published:** 2024-03-12

**Authors:** Yiran Zhang, Junni Zhang, Pooria Lesani, Zufu Lu, Hala Zreiqat

**Affiliations:** Tissue Engineering & Biomaterials Research Unit, School of Biomedical Engineering, Faculty of Engineering and IT, The University of Sydney, Darlington, NSW 2006 Australia

**Keywords:** Ageing, Osteopontin, Stem cells, Bone, Senescence, Cell shape and morphology

## Abstract

The regenerative function of stem cells is compromised when the proportion of senescent stem cells increases with ageing advance. Therefore, combating stem cell senescence is of great importance for stem cell-based tissue engineering in the elderly, but remains largely unexplored. Osteopontin (OPN), a glycosylated phosphoprotein, is one of the key extracellular matrix molecules in bone tissue. OPN activates various signalling pathways and modulates cellular activities, including cell senescence. However, the role of OPN in stem cell senescence remains largely unknown. This study aims to investigate if OPN modulates cell senescence and bone regenerative function in human adipose-derived mesenchymal stem cells (ASCs), and to determine the underlying mechanisms. We first developed a senescent ASC model using serial passaging until passage 10 (P10), in which senescent cells were characterised by reduced proliferation and osteogenic differentiation capacity compared to P4 ASCs. The conditioned medium from P10 ASCs exhibited a diminished trophic effect on human osteoblasts (HOBs), compared to that from P4 ASCs. P10 ASCs on OPN-coated surface showed rejuvenated phenotype and enhanced osteogenic differentiation. The conditioned medium from P10 ASCs on OPN-coating improved trophic effects on HOBs. OPN regulated the morphology of senescent ASCs, transforming them from a more rounded and flattened cell shape to an elongated shape with a smaller area. These findings demonstrated the effects of OPN in restoring senescent ASCs functions, possibly through a mechanism that involves the modulation of cell morphology, indicating that OPN might hold a great potential for rejuvenating senescent stem cells and could potentially open a new venue for regenerating bone tissue in age-related diseases.

**Graphical Abstract:**

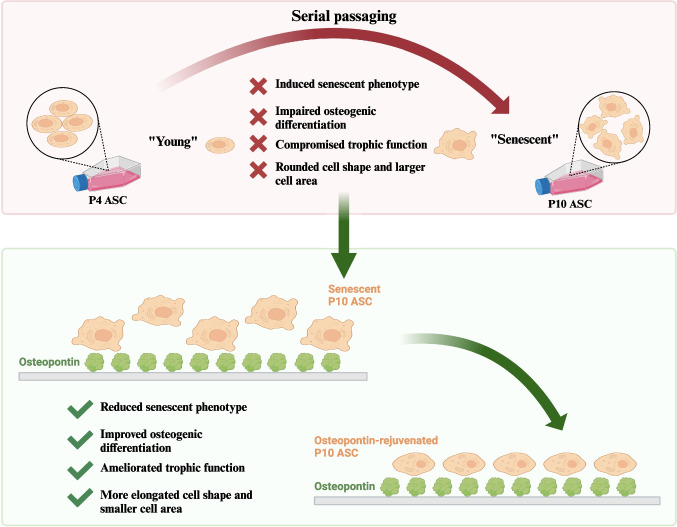

**Supplementary Information:**

The online version contains supplementary material available at 10.1007/s12015-024-10707-5.

## Introduction

With the ongoing increment in human life expectancy, countries worldwide are grappling with rapid growth of the ageing population, consequently, escalating the prevalence of age-related social and medical problems [1–3]. The World Health Organisation (WHO) projected a doubling of the population for individuals aged over 60 and 80 years old between 2020 and 2050. As individuals age, their physiological capacity and functionality are gradually lost, leading to a higher risk of health conditions such as musculoskeletal diseases [2].

Bone is a complex tissue that serves a significant role in the human body. The maintenance of bone mineral density and resistance to fracture is controlled by the coordination of several cell mature types: osteoblasts, osteoclasts, and osteocytes. Despite the excellent regenerative capacity of bone tissue in response to injury, there is a decline in the regenerative performance of bone tissue in the elderly. While the implantation of bone autografts is considered the gold standard for repairing and regenerating large bone defects, it has significant drawbacks, including limited graft supply and risk of donor site morbidity. These challenges have prompted scientists to explore alternative solutions to conventional bone autograft treatments for bone tissue regeneration.

Mesenchymal stem cells (MSCs) have been attracting extensive attention from scientists for tissue engineering applications due to their unique properties. These include the release of trophic factors, immunomodulation capacity, and the ability to differentiate into diverse cell lineages, including the osteoblast lineage [4]. The remarkable self-renewal and osteogenic differentiation capacity of MSCs render them a promising candidate for bone tissue regeneration [5, 6]. Beyond their isolation of MSCs from bone marrow back in 1960s, MSCs have been successfully derived from many other tissues, including adipose tissue [7]. Adipose-derived stem cells (ASCs) have been extensively used in bone tissue regeneration, as they offer significant advantages over MSCs, including the abundance of adipose tissue, less invasive procedure to obtain, and a higher yield of cells [7]. ASCs have also been used in combination with scaffolds for bone tissue engineering in vivo due to their outstanding proliferation rate, their ability to release trophic factors within recipient site tissue, and desirable differentiation capacity compared to other MSC types [5–7]. Despite the promising perspective of ASCs for bone tissue regeneration, inconsistencies remain in the efficacy of the ASC-based tissue engineering approach for tissue repair and regeneration. Current knowledge indicates that the age and morbidity state of cell donors critically influence the quantity and quality of isolated MSCs. For instance, the increased presence of senescent MSCs with age or extensive MSC expansion in vitro would result in the diminished osteogenic differentiation potential of MSCs [8, 9].

Cell senescence denotes a state of altered cellular function characterised by permanent arrest in cell cycle growth. It can occur during in vitro cell proliferation and expansion, as well as through the chorological ageing process [10, 11]. The concept of cell senescence was first introduced in the 1960s by Hayflick who discovered that human cells have a limited capacity for replication, after which they become senescent. This finding initiated the broader discussion on the concept of cell senescence [12]. Cell senescence can be triggered by a variety of stimuli, including DNA damage, oxidative stress, and oncogenic activation. The primary mechanism linked to ageing, particularly in the elderly, is replicative senescence. This process involves the shortening of the telomeres at chromosome ends during DNA replication, due to the dysfunction of DNA polymerase at the single-stranded 3’ end. This shortening is commonly regarded as an ageing indicator. Replicative senescence occurs when telomeres reach a critically short length, leading to the halting of the cell cycle growth [13]. In addition to this growth arrest, cell senescence is also characterised by a senescence-associated secretory phenotype (SASP). SASP factors are known to contribute to inflammatory responses in the surrounding tissue, revealing the paracrine role of senescent cells [14]. Consequently, cell senescence plays a causative role in the reduction of tissue functionality and regenerative capacity as part of the ageing process [15]. Therefore, it is crucial to develop strategies for rejuvenating senescent MSCs and restoring their functionality, particularly in elderly patients in need of bone tissue engineering solutions.

Human tissues are characterised by highly ordered nanostructures and chemical composition that present coordinated signals to resident cells. Cell functionality is, therefore, tightly regulated by cues from the tissue niche, which include both biochemical and physical signals [16]. Our previous research demonstrated that synthetic biomaterials could interact with senescent human osteoblasts, creating an anti-senescent microenvironment that promotes bone regeneration in aged animals [17]. This finding suggests that introducing suitable biomaterials might be an effective strategy for the rejuvenation of senescent ASCs. Osteopontin (OPN), a phosphoprotein of the extracellular matrix (ECM), was first identified in the 1980s as a secreted protein in bone. It is involved in numerous physiological and pathological processes, including bone tissue remodelling, wound healing, inflammation, and cancer [18, 19]. OPN plays a crucial role in various age-related diseases, such as non-alcoholic fatty liver disease and cardiac fibrosis [18, 20]. Supplementation of OPN in the extracellular microenvironment exerts ameliorative influences on the proliferation and differentiation capacity of human bone marrow MSCs in a dose-dependent manner [21]. Considering that OPN, a key non-collagenous component in bone ECM, gradually decreases with age advancing [22], we hypothesise in this study that OPN might play a role in modulating the senescent phenotype of ASCs and their osteogenic regenerative capacity. The aim of this study is to explore the effects of OPN on the senescent phenotype of ASCs and to determine its fundamental mechanisms in modulating the senescence and bone regeneration capabilities of ASCs.

## Materials and Methods

### Cell Culturing and Seeding

ASCs were purchased from Thermo Fisher Scientific Inc. The cells were cultured at 37℃ with 5% CO_2_ in culture medium. The growth medium consisted of Minimal Essential Medium-α (MEM-α), 10% Fetal Bovine Serum (FBS), and 1% 30 mg/mL penicillin and 100 mg/mL streptomycin (Gibco Laboratories), while the osteogenic medium was made up of StemPro®, StemPro® Osteogenesis Supplement (Thermo Fisher Scientific Inc.), and 1% 30 mg/mL penicillin and 100 mg/mL streptomycin. The culture medium was refreshed every 3 days until the cell confluence reached around 80% to 90%. Once the confluence was achieved, the cells were detached by TrypLE™ (Gibco Laboratories) and centrifuged at 1300 rpm for 5 min to obtain the cell pellet. After removing the supernatant of the centrifuged cell suspension, ASCs were resuspended in the growth medium for serial passaging. ASCs at Passage 4 (early passaged) and Passage 10 (late passaged) were used for experiments in this study.

The cells were seeded at a density of ~ $1\times {10}^{5}$ cells/well and ~$1\times {10}^{4}$ cells/well in 12-well plates and 96-well plates for gene expression experiments and cell proliferation assays, respectively. For staining experiments, the ASCs were seeded at a density of ~ 12,000 cells/well in 24-well plates. Once seeded in the well plates, the ASCs were incubated at 37℃ with 5% CO_2_ for cell attachment and proliferation. All cells were seeded with four replicates.

### Preparation of OPN-Coating

Human OPN (Sigma-Aldrich, USA) were coated on cell culture plates (NUNC) by adding OPN at various concentrations (0.04, 0.2, 1, and 5 μg/ml) in phosphate-buffered saline (PBS) and being incubated for 2 h at 37 °C. The wells were then extensively rinsed by washing three times with PBS and then being incubated with 3% bovine serum albumin (BSA; Sigma-Aldrich, USA) in PBS for 1 h at 37 °C to block nonspecific protein binding sites. The wells in control group were treated only with 3% BSA for 1 h at 37 °C.

### Real-Time PCR

The isolation of RNA was completed using RNeasy® Mini Kit (Qiagen, Germany) according to the accompanied protocol. Based on the extracted RNA concentration, 1 μg RNA was obtained to synthesize complementary DNA (cDNA) using SensiFAST™ cDNA Synthesis Kit (Bioline, MA). The synthesized cDNA was then utilized as a template to conduct real-time quantitative polymerase chain reaction (qPCR) in a PCR machine (Rotor-Gene Q; Qiagen, Germany) using the primers as described in a previous study [17]. qPCR used SYBR® Green as the fluorescent dye and completed 45 cycles of 95℃ denaturation, 60℃ primer annealing, and 72℃ extension and detection. The relative mRNA expressions of cell cycle regulators (*P16, P21,* and *P53*), SASP factors (*IL-6, IL-1β,* and *TNF-α*), and osteogenic differentiation regulators (*Runx2, osteopontin, BMP-2,* and *osteocalcin*) were normalised to the gene expression of the housekeeping gene *18s*.

### Cell Proliferation Assay

The proliferative capacity of cells was assessed using Cell Proliferation ELISA, BrdU (colorimetric) Kit (Roche Applied Science, Indianapolis, IN). Briefly, cells were seeded at a density of ~ 10,000 cells/well in 96-well plates and labelled by 100 μM BrdU overnight at 37℃ with 5% CO_2_. After removing the labelling solution, FixDenant was added to fix the cells. The antibody conjugate, Anti-BrdU-POD solution was then used to bind the BrdU incorporated in the newly synthesized DNA, and the cells were incubated for 90 min at room temperature. Cells were then washed three times with PBS to remove excess antibody, and the substrate solution was added for a 30-min incubation at 25℃ for colour development. The quantification of cell proliferation was performed by measuring the absorbance at 370 nm using a Multiskan Spectrophotometer (Thermo Fisher Scientific Inc.).

### Senescence-Associated Beta-Galactosidase (SA-β-Gal) Assay

The activity of SA-β-Gal was examined using a Senescence Cells Histochemical Staining Kit (Sigma-Aldrich, USA). Briefly, the cells were washed twice with PBS after the removal of culture media and fixed by Fixation buffer for 10 min at room temperature. Upon removal of Fixation buffer, the freshly made staining solution was added according to the manufacturer’s instructions. The cells were then kept in dark and incubated at 37℃ without CO_2_ until cells were stained blue. The light microscopy was utilised for the visualization of SA-β-Gal positive (blue) cells and at least 10 images were captured from each group for quantification. The SA-β-Gal positive cells were counted and represented as a percentage of total cell numbers in the images and the mean values for each experimental group were obtained.

### Alizarin Red Staining Assay

To measure the mineralisation of cells, Alizarin Red purchased from Sigma-Aldrich was used for staining. The culture media were removed by aspiration. For the fixation of cells, 70% ethanol was added after washed the cells once with PBS, followed by a 10-min incubation at room temperature. Cells were washed twice with ddH_2_O after removal of ethanol. The cells were then stained with filtered Alizarin Red solution at pH 4.2 for 30 min in dark at room temperature. The red positive mineralised area could be observed under microscope. A quantitative analysis was conducted using ImageJ (National Institutes of Health, Bethesda, MD, USA), an image analysis software, to obtain the percentage of stained area over the captured image.

### Conditioned Media Collection and Human Osteoblasts (HOBs) Culturing

ASCs at P4 or P10 were grown at different conditions, including tissue culture plastic, OPN-coated surface and Y27632 treatment. When cells were at 80% to 90% confluence, culture media were removed, and then washed three times with phosphate buffered saline (PBS) and twice with serum-free α-MEM media. Serum-free α-MEM media were added to cells to be conditioned at 37°C for 48 h. Conditioned media were then collected. Detached cells and debris were removed from conditioned media by passing through a 0.22 μm filter and stored at − 80°C until use.

HOBs were isolated from the discarded human bone tissue from the patients, and the permission was granted by the Sydney Children's Hospitals Network Human Research Ethics Committee and informed consent was obtained. Human trabecular bone was used for isolating HOBs as described previously [23]. The cells were cultured at 37°C with 5% CO_2_, and culture medium was changed every 3 days until cells were passaged at 80% to 90% confluence. All HOBs at passage 3 used in the experiments were the cell mixtures sourced from three independent donors (2 males and 1 female, teenagers).

### Phalloidin Staining, Cell Shape and Size Analysis

ASCs were grown in the wells of 48-well clear bottom plate cultured in different conditions. At the designated time points, cell culture medium was aspirated, and the cells were washed three times with PBS. Then the cells were fixed in freshly prepared 4% formaldehyde in PBS at room temperature for 30 min. Aspirate fixation solution and wash cells 3 times with PBS. Add 0.1% Triton X-100 in PBS into the fixed cells for 5 min to increase cellular permeability, and then wash cells 3 times with PBS, followed by the addition of phalloidin-conjugate working solution (P5282) and incubation at room temperature for 1 h. Rinse cells 3 times with PBS and add DAPI dye for nuclei staining. After another 3 times of rinses with PBS, the cell images were visualized and analysed using Invitrogen EVOS M5000 Cell Imaging System (Thermo Fisher Scientific Inc.).

### Statistical Analysis

The data were obtained from four replicates and represented with mean ± SE. Statistical SPSS 24 was used for data analysis and the figures were drawn using GraphPad Prism. Levene's and ANOVA test were used to analyse data between two groups and three or more treatment groups, respectively. Group differences were considered significant if *P* < 0.05.

## Results

### Serial Cell Passaging Induced Cell Senescence and Compromised the Regenerative Function in ASCs

**Fig. 1:**
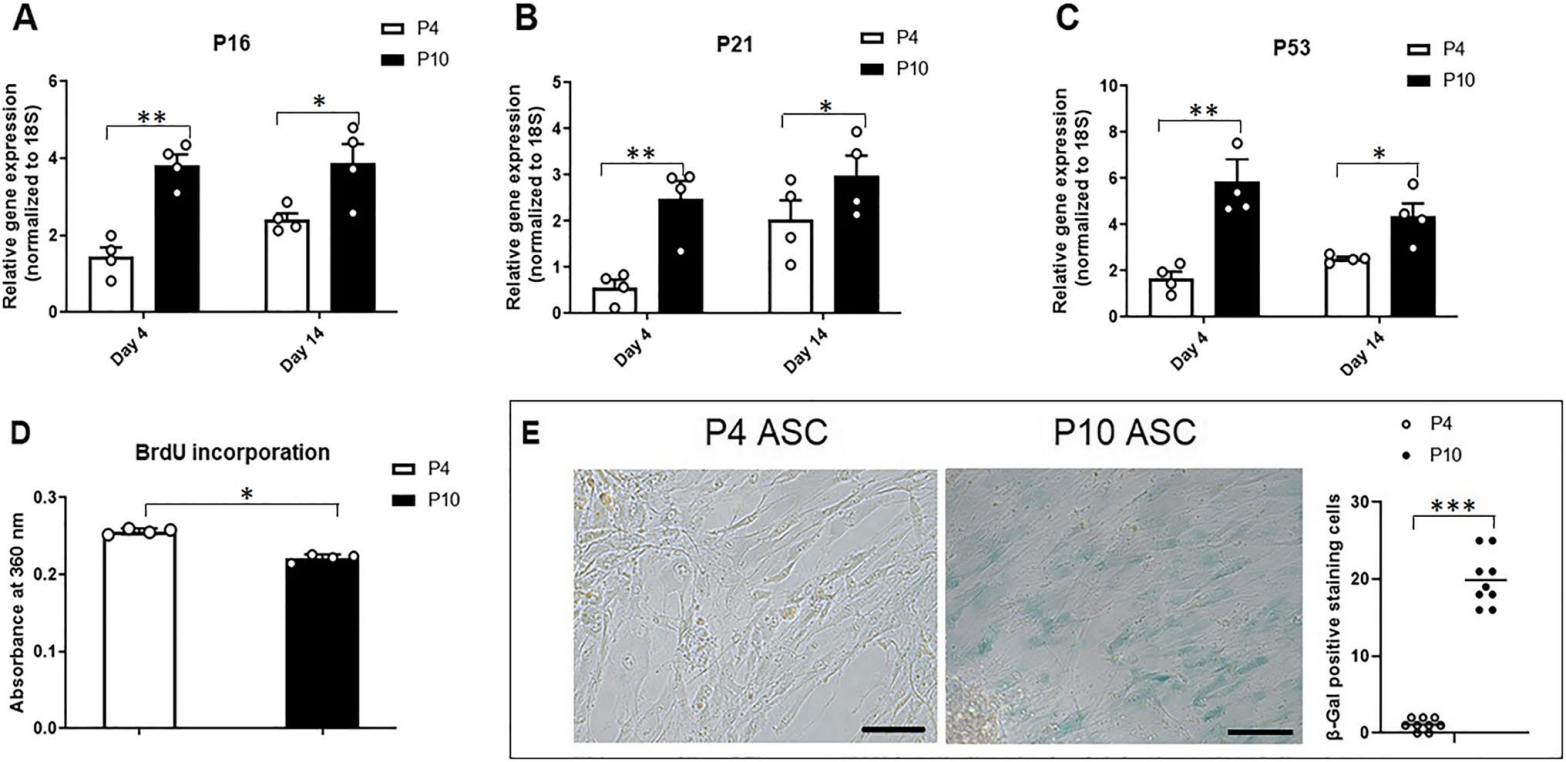
**Serial cell passaging induced senescent phenotype in ASCs.** Cell senescence-associated markers were assessed and compared in ASCs at passage 4 and 10. (**A**-**C**) At both Day 4 and Day 14, the cell cycle regulation-associated genes (*P16, P21,* and *P53*) in P10 ASCs were significantly increased compared to ASCs at P4. (**D**) The proliferative capacity of P10 ASCs was reduced in comparison to P4 ASCs. (**E**) After 72 h of cell culture, the observed percentage of SA-β-Gal positive staining cells was higher in P10 ASCs (20%) than that in P4 ASCs (2%). *: *P* < 0.05, **: *P* < 0.01, ***: *P* < 0.001

Cell passaging has been widely used to induce cell senescence in primary cells, simulating the natural process where mitotically competent cells cease to proliferate in vivo [24, 25]. We first established an in vitro model of replicative cell senescence in human ASCs through serial cell passaging. We found that mRNA levels of cell senescence-associated cell cycle regulation genes (*P16, P21* and *P53)* in P10 ASCs were significantly higher than those in P4 ASCs (Fig. 1A-C). In addition, ASCs’ proliferation in P10 ASCs was significantly lower than that in P4 ASCs after 72 h of cell seeding (Fig. 1D), and P10 ASCs displayed a significantly higher percentage (20%) of SA-β-Gal positive staining cells, compared to P4 ASCs (2%) (Fig. 1E, full-size image Fig. S1).

### Serial Cell Passaging Impaired Osteogenic Differentiation Ability in ASCs

**Fig. 2:**
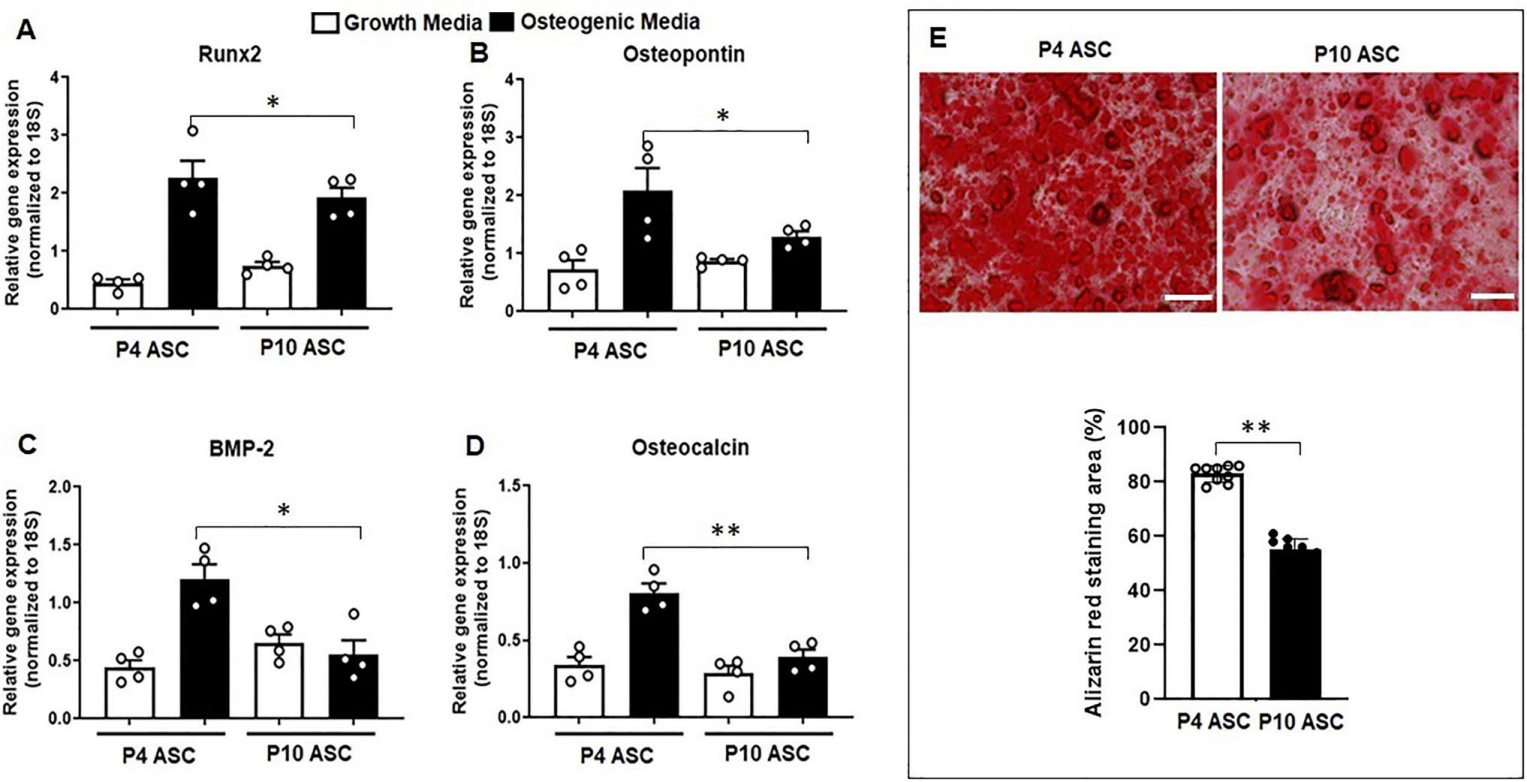
**Serial cell passaging compromised osteogenic differentiation ability in ASCs.** Osteogenic differentiation-associated markers were examined and compared in ASCs at different passages (P4 and P10) cultured in both growth media and osteogenic media. (**A**-**D**) Expressions of osteogenic genes (*Runx2, osteopontin, BMP-2,* and *osteocalcin*) in ASCs cultured in osteogenic media were increased compared to ASCs cultured in growth media. The expressed mRNA levels of osteogenic genes in P10 ASCs were downregulated compared to P4 ASCs. (E) After being cultured in osteogenic media for 21 days, the Alizarin Red staining illustrated a significant decrease in mineralisation. *: *P* < 0.05, **: *P* < 0.01, ***: *P* < 0.001

As human age, an increase in the accumulation of senescent cells within the tissue contributes to impaired osteogenic differentiation in stem cells [8]. To explore whether serial cell passaging affects the osteogenic potential of senescent ASCs, we assessed the mRNA expression levels of osteogenesis-related genes (*Runx2, osteopontin, BMP-2,* and *osteocalcin*) in both P4 and P10 ASCs cultured in growth media and in osteogenic media, respectively. No significant difference in osteogenic gene expressions (*Runx2, osteopontin, BMP-2,* and *osteocalcin*) was observed between P4 and P10 ASCs when cultured in growth media. However, when P10 ASCs were cultured in osteogenic media, they exhibited significantly lower levels of these genes (*Runx2, osteopontin, BMP-2, and osteocalcin*) compared to P4 ASCs cultured in the same osteogenic media (Fig. 2A-D). Additionally, the area stained with Alizarin Red in P10 ASCs at Day 21 was smaller than that in P4 ASCs (Fig. 2E, full-size image Fig. S2). This suggests serial passaging ASCs results in a decreased capacity for bone formation.

### Trophic Function on HOBs was Diminished in Serial Passaged ASCs

**Fig. 3:**
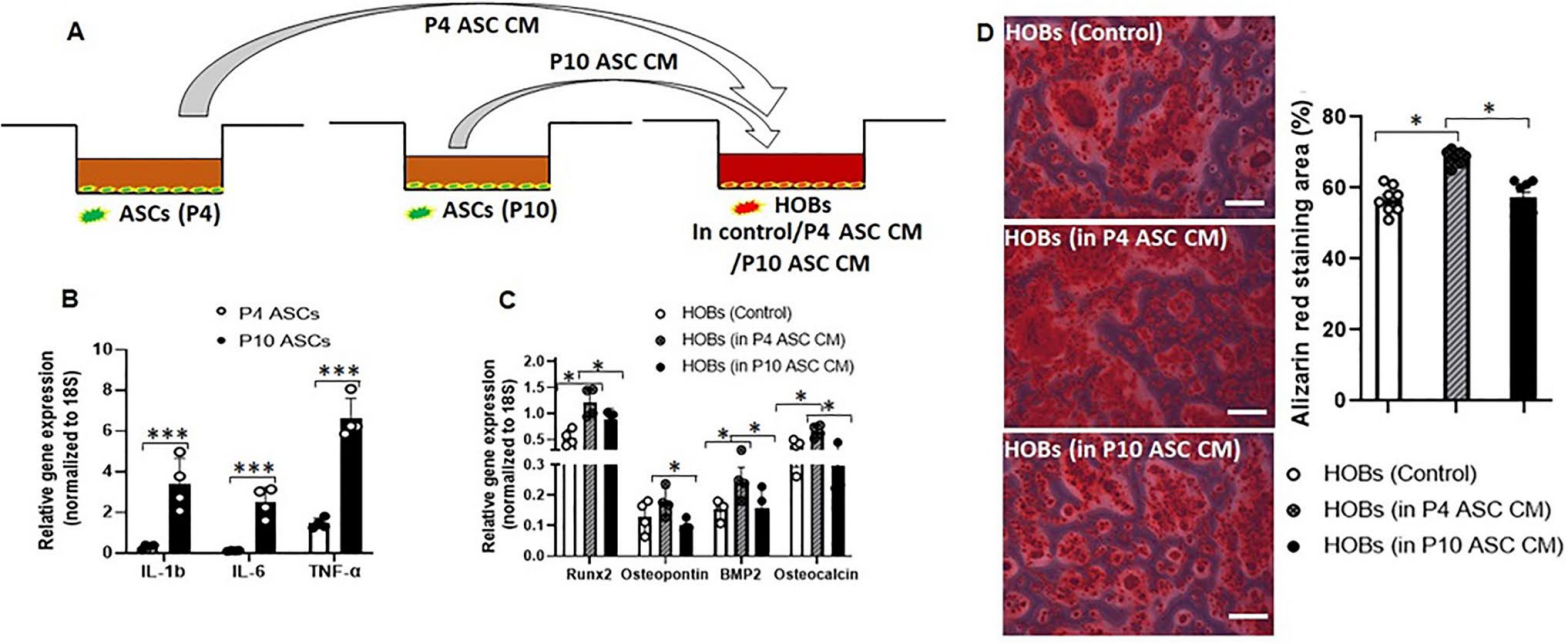
**P10 ASCs showed diminished trophic function on HOBs.** (**A**) A schematic illustration of conditioned media collection. Conditioned media (CM) were collected from both P4 and P10 ASCs and used to culture HOBs. The control group used normal growth media to culture HOBs. (**B**) Normalised mRNA expression levels of SASP factors (*IL-1β, IL-6,* and *TNF-α*). (**C**) HOBs cultured in P10 ASCs CM showed diminished expression of osteogenic genes (*Runx2, osteopontin, BMP-2,* and *osteocalcin*) in comparison to those cultured in P4 ASCs CM. (**D**) The Alizarin Red staining area of HOBs cultured in P10 ASCs CM is significantly smaller than those cultured in P4 ASCs CM. *: *P* < 0.05, **: *P* < 0.01, ***: *P* < 0.001

ASCs are known for their trophic functions, influencing neighbouring cells in the local microenvironment and modulating tissue regeneration through the secretion of trophic factors [26]. To study the influences of serial cell passaging on the trophic functions (SASP factors) of ASCs, we collected the culture media from P4 or P10 ASCs, termed as conditioned media (CM), to culture HOBs (Fig. 3A), and compared the osteogenic genes (*Runx2, osteopontin, BMP-2,* and *osteocalcin*) expressed from HOBs cultured in CM, with those from HOBs grown in normal growth media. As shown in Fig. 3B, mRNA expression levels of SASP factors (*IL-1β, IL-6,* and *TNF-α*) in P10 ASCs were significantly higher than those in P4 ASCs. The increased expression of SASP factors correlated with the induced senescent phenotypes resulting from serial passaging in ACSs (Fig. 1A-E).

Moreover, we investigated the impact of the CM from ASCs on the osteogenic differentiation ability of HOBs. Interestingly, when HOBs were cultured in CM from P4 ASCs, as opposed to growth media, there was a significant increase in mRNA levels of osteogenic genes (*Runx2, osteopontin, BMP-2, and osteocalcin*). This suggests that P4 ASCs exert trophic effects on HOBs through paracrine signalling (Fig. 3C). However, the trophic function on HOBs was compromised when cultured in P10 ASCs CM (Fig. 3C). As in comparison with the CM from P4 ASCs, the gene expression levels of *Runx2, osteopontin, BMP-2,* and *osteocalcin* in HOBs were downregulated when they were cultured in CM from P10 ASCs. The same trend was observed in Alizarin Red staining: HOBs cultured in CM from P4 ASCs exhibited increased staining, whereas HOBs cultured in CM from P10 ASCs did not show this enhancement compared to those grown in normal growth media (Fig. 3D, full-size image Fig. S3).

### Osteopontin Rejuvenated Senescent ASCs and Enhanced Their Osteogenic Regenerative Function

**Fig. 4:**
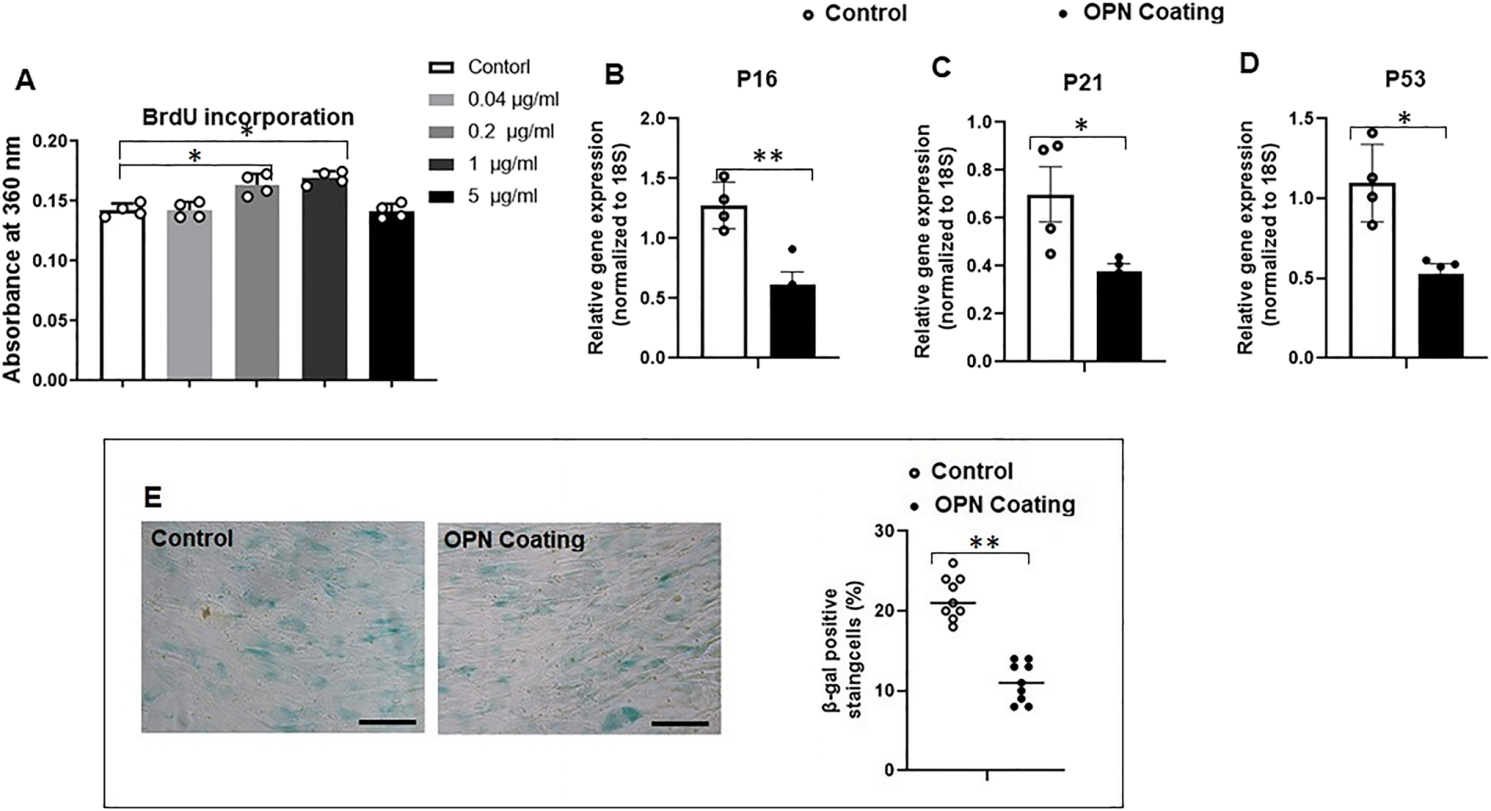
**Osteopontin rejuvenated senescent phenotype of P10 ASCs.** Cell senescence-associated markers were assessed and compared in P10 ASCs cultured on tissue culture substrate and OPN-coated substrate. (**A**) P10 ASCs were cultured with a concentration gradient of OPN-coating (control, 0.04 μg/mL, 0.2 μg/mL, 1 μg/mL, and 5 μg/mL), and the absorbance at 370 nm were measured to reflect the cell proliferation at different concentrations. OPN-coating at 1 μg/mL exhibited the optimal proliferative ability of P10 ASCs. (**B**-**D**) The cell cycle regulation-associated genes (*P16, P21,* and *P53*) in P10 ASCs cultured on OPN-coating were significantly reduced compared to the control. (E) After 72 h of cell seeding, the percentage of SA-β-Gal positive (blue) cells was reduced in OPN group (12%) in comparison to that in control (22%). *: *P* < 0.05, **: *P* < 0.01, ***: *P* < 0.001

After successfully inducing a senescence in ASCs and confirming their diminished osteogenic regenerative potentials, we then investigated how OPN could impact the senescence characterization and osteogenic regenerative function in the senescent ASCs. To this aim, senescent P10 ASCs were cultured on tissue culture plates coated with various concentrations of OPN (0.04, 0.2, 1, and 5 µg/ml) and their cell proliferation was investigated (Fig. 4A). At a concentration of 0.04 µg/ml, OPN-coating did not significantly affect cell proliferation in P10 ASCs. However, as the concentration increased to 0.2 and 1 µg/ml, OPN-coating promoted cell proliferation in P10 ASCs. This effect was not observed at the higher concentration of 5 µg/ml. Therefore, we chose 1 µg/ml of OPN-coating for subsequent studies on senescent phenotype and regenerative characteristics. For senescent cell phenotype characterization, there was a significant reduction in the mRNA expressions of cell cycle regulators (*P16, P21,* and *P53*) in P10 ASCs seeded on substrate coated with 1 μg/mL OPN, compared to those cultured on control substrate without OPN-coating for 14 days (Fig. 4B-D). Additionally, after 72 h of cell culture, the percentage of SA-β-Gal positive (blue) cells in P10 ASCs was significantly lower when OPN-coating was present (12%), compared to the control (22%) (Fig. 4E, full-size image Fig. S4). Our results suggest that OPN-coating can rejuvenate senescent ASCs, as it positively influences their proliferation and reduces markers of senescence, ultimately enhancing their regenerative potential.

**Fig. 5:**
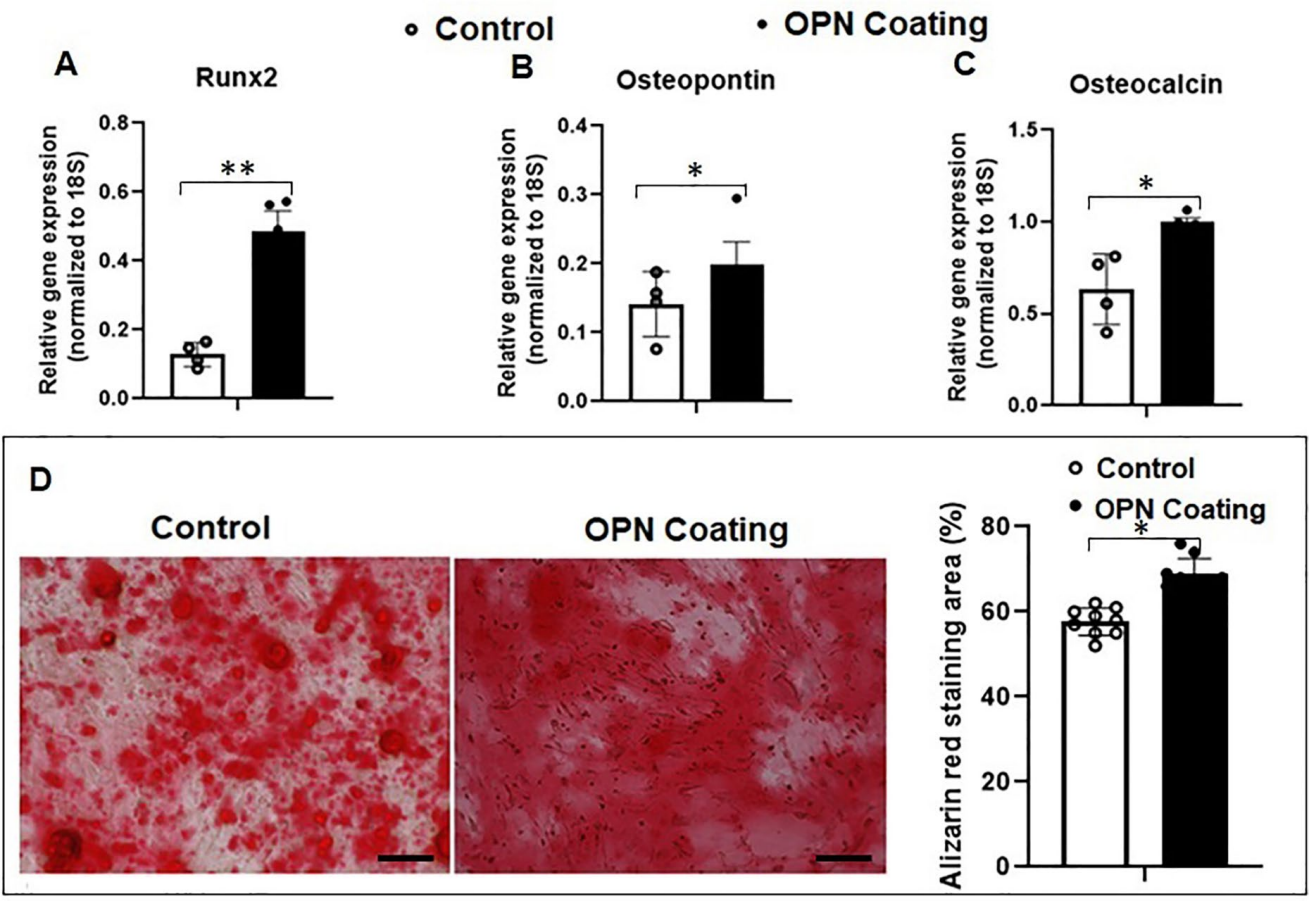
**Osteopontin-coating improved osteogenic differentiation of P10 ASCs.** Osteogenesis-associated mRNA levels were measured in P10 ASCs cultured on the substrates with or without OPN-coating. (**A**-**C**) Expressions of osteogenic genes (*Runx2, osteopontin,* and *osteocalcin*) in P10 ASCs cultured on OPN-coating were significantly upregulated compared to the control. (**D**) After culturing of 21 days, the Alizarin Red staining area in OPN group was increased compared to the P10 ASCs in the control. *: *P* < 0.05, **: *P* < 0.01, ***: *P* < 0.001

We then investigated how OPN could potentially restore the osteogenic differentiation ability of senescent ASCs when cultured in osteogenic media. Our results showed that P10 ASCs cultured on OPN-coated surfaces exhibited significantly higher mRNA levels of osteogenic genes (*Runx2, osteopontin,* and *osteocalcin*) in contrast to P10 ASCs on the substrate without OPN-coating (Fig. 5A-C). In addition, after 21 days of culturing, the mineralisation of P10 ASCs on OPN-coated surfaces was also enhanced, as evident by the larger Alizarin Red positive area (Fig. 5D, full-size image Fig. S5). Therefore, our results indicate that OPN has a positive effect on revitalising the osteogenic differentiation potential of senescent ASCs.

**Fig. 6:**
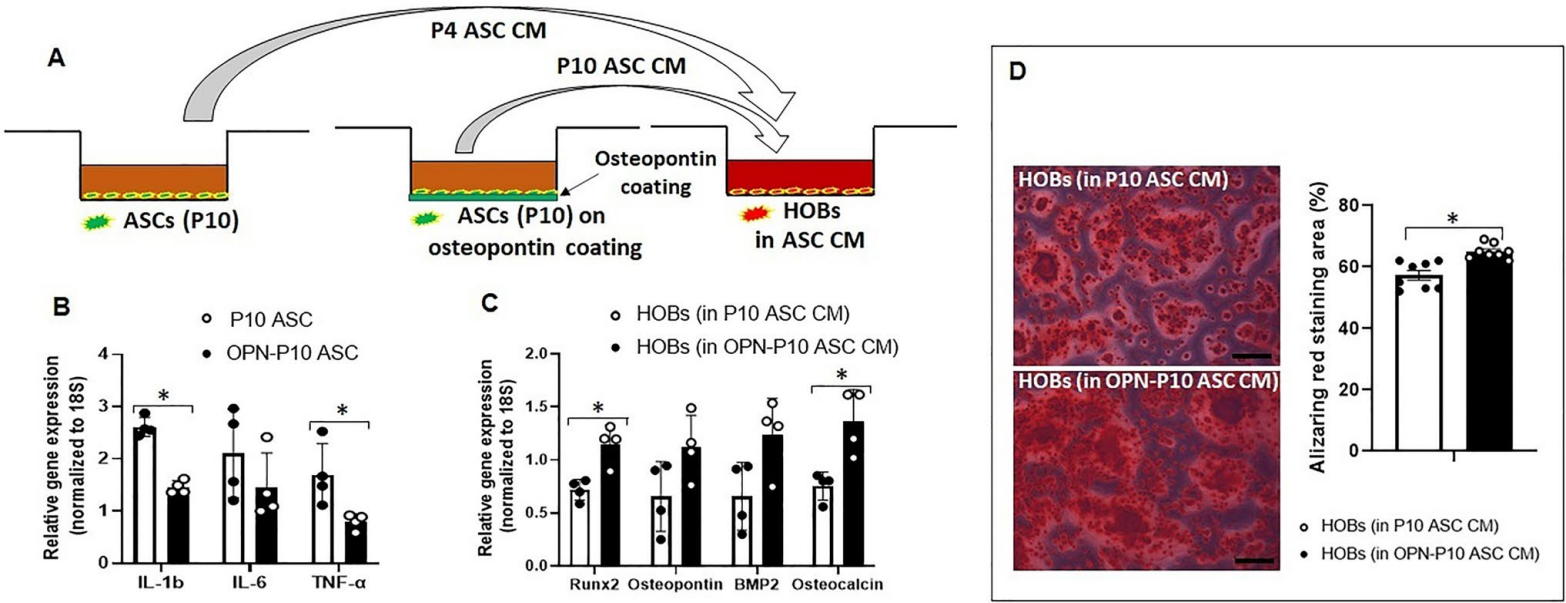
**P10 ASCs on osteopontin-coating showed enhanced trophic function on HOBs.** (**A**) A schematic illustration of conditioned media collection. Conditioned media (CM) were collected from P10 ASCs cultured on cell culture plates with or without OPN-coating and then used to culture HOBs. (**B**) Normalised mRNA expression levels of SASP factors (*IL-1β, IL-6,* and *TNF-α*) in P10 ASCs on OPN-coating were significantly reduced in contrast to those cultured on cell culture plates. (**C**) HOBs cultured in OPN-P10 ASCs CM presented rejuvenated expressions of osteogenic genes (*Runx2* and *osteocalcin*) in comparison to those cultured in P10 ASCs CM. (**D**) The Alizarin Red staining area of HOBs cultured in OPN-P10 ASCs CM implied a restored calcification than those cultured in P10 ASCs CM. *: *P* < 0.05, **: *P* < 0.01, ***: *P* < 0.001

In addition, we sought to investigate whether OPN could regulate the trophic function of stem cells by modulating the osteogenic differentiation of HOBs. To do so, we collected the CM from P10 ASCs cultured on the well plates with or without OPN-coating and examined their trophic function on HOBs (Fig. 6A). Prior to assessing the effects of CM on HOBs, we measured the mRNA expressions of SASP factors (*IL-1β, IL-6,* and *TNF-α*) in P10 ASCs and found significant downregulation when P10 ASCs were cultured on OPN-coated surface (Fig. 6B). Besides, HOBs cultured in CM from P10 ASCs on OPN-coated surfaces exhibited significantly higher mRNA expression levels of osteogenic genes, including *Runx2* and *osteocalcin* (Fig. 6C). The Alizarin Red staining area aligned with osteogenic gene expressions, indicating that the CM from P10 ASCs enhanced the mineralisation ability of HOBs (Fig. 6D, full-size image Fig. S6).

### Osteopontin Rejuvenated Senescent ASCs Through Modulating Cell Shape

**Fig. 7:**
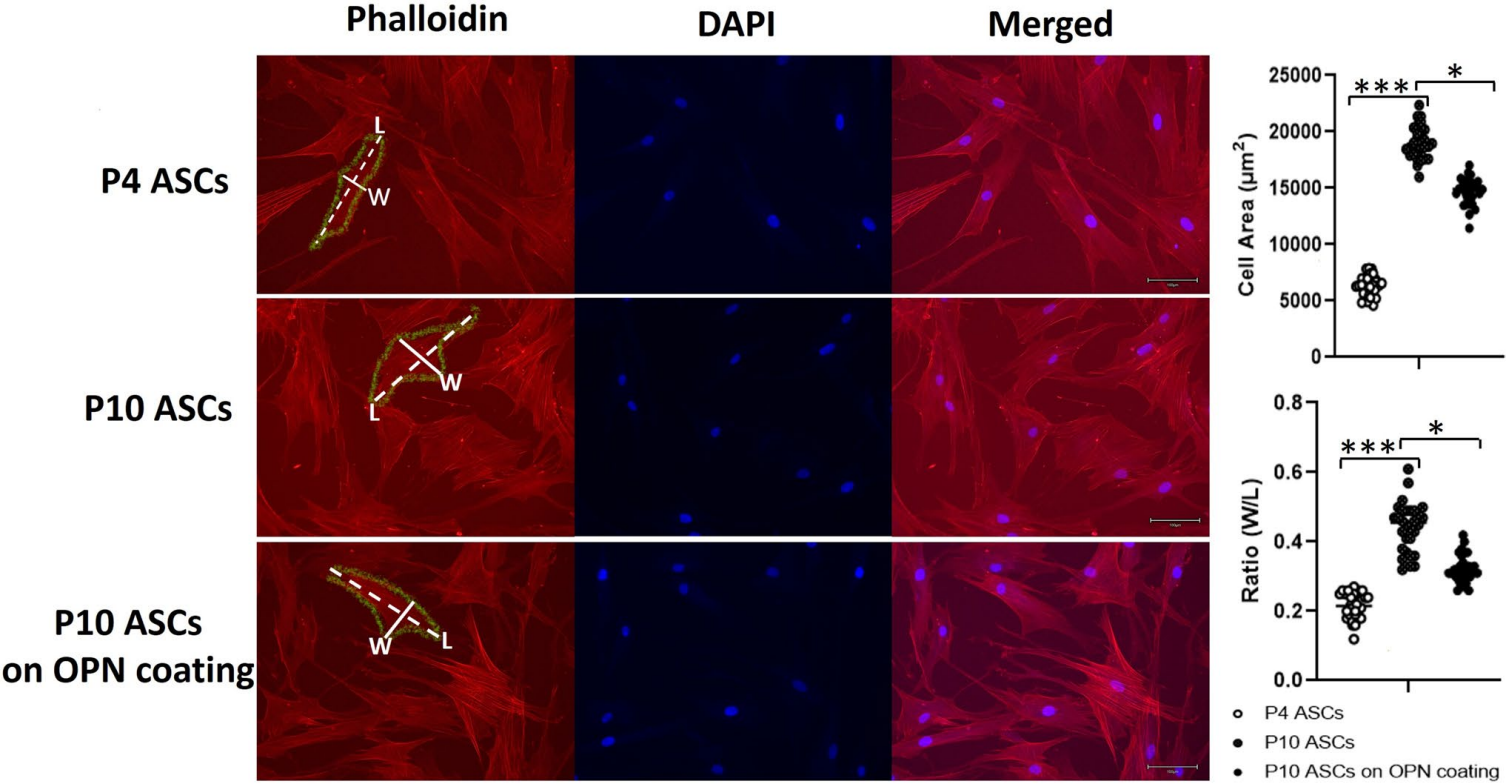
**Osteopontin modulated the cell shape of P10 ASCs.** Immunofluorescence staining of P4 ASCs, P10 ASCs, and P10 ASCs on OPN-coating. Phalloidin was used to stain the cytoskeleton of ASCs. The nuclei were stained blue with DAPI. The representative measurements of cell width (W) and length (L) were indicated by the solid line and the dashed line, respectively. The cell area of P10 ASC was larger than that of P4 ASC. When culturing P10 ASCs on OPN-coating, the cell area became smaller than those cultured on tissue culture plastic. P10 ASCs displayed an increased width to length (W/L) ratio in comparison to P4 ASCs, whereas OPN exerted a modulatory effect on P10 ASCs to reduce the W/L ratio. *: *P* < 0.05, **: *P* < 0.01, ***: *P* < 0.001

Morphological changes, including alterations in cell size and shape, represent critical characteristics and potential underlying mechanisms of cell senescence [27–29]. To assess whether OPN could rejuvenate the effect of senescence in ASCs by modulating cell morphology, we visualised the cytoskeleton of P4 ASCs, P10 ASCs, and P10 ASCs on OPN-coating to measure the differences in their cell area and shape (Fig. 7, full-size image Fig. S7). Comparing P4 ASCs and P10 ASCs, we observed that the cell area of P10 ASCs was significantly larger than that of P4 ASCs. Similarly, the ratio between width and length (W/L) of P10 ASCs was larger than P4 ASCs. Interestingly, when cultured on OPN-coating, the enlarged cell area of P10 ASCs was reduced, as was the W/L ratio. These results suggest that OPN may rejuvenate senescent ASCs by modulating their cell morphology.

**Fig. 8:**
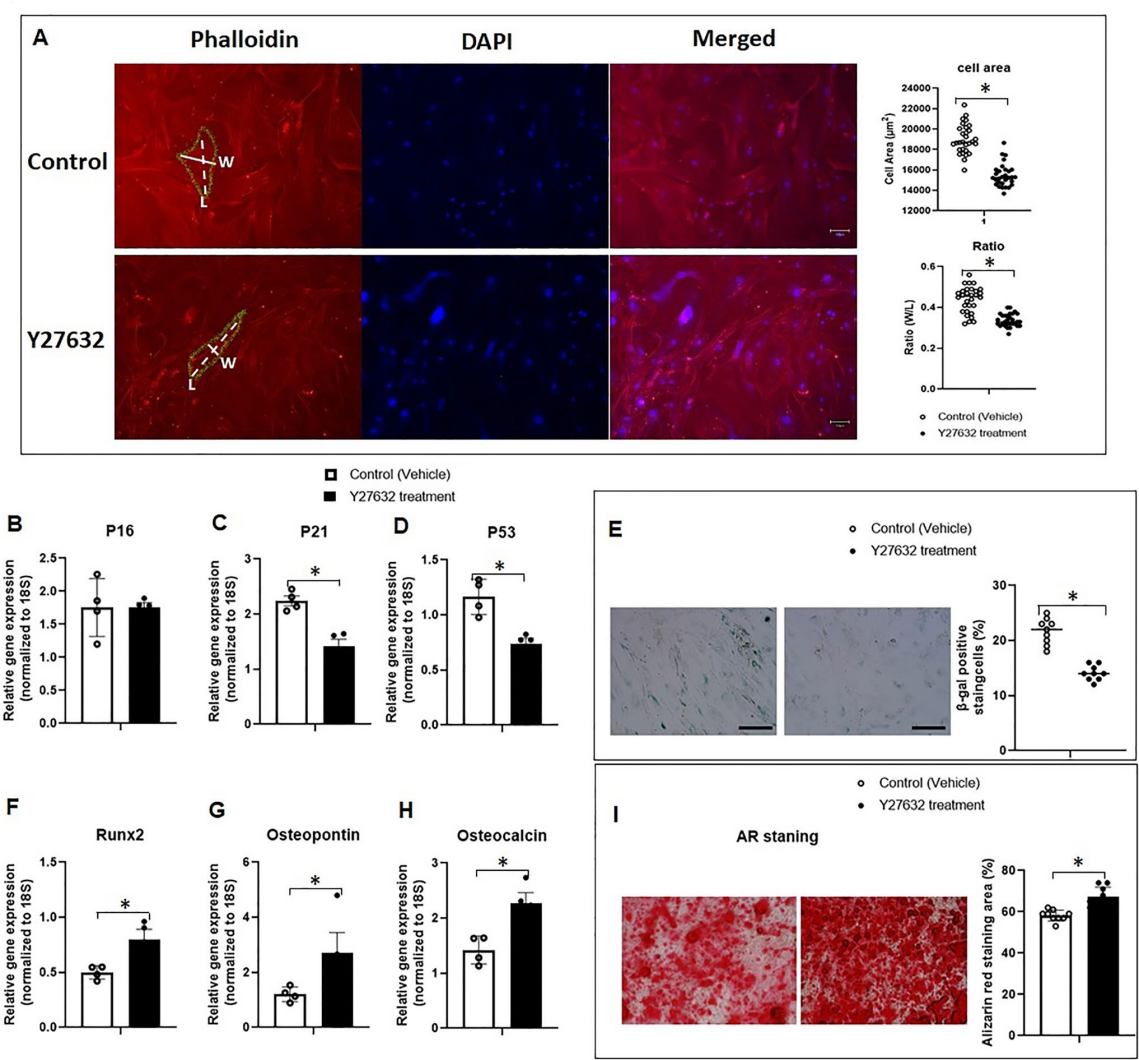
**Y27632 rejuvenated the cell shape and senescent phenotypes of P10 ASCs.** (**A**) Immunofluorescence staining of P10 ASCs treated with and without Y27632. Phalloidin was used to stain the cytoskeleton of ASCs. The nuclei were stained blue with DAPI. The representative measurements of cell width (W) and length (L) were indicated by the solid line and the dashed line, respectively. The cell area and W/L ratio of Y27632-treated P10 ASCs were significantly reduced in contrast to the control. (**B**-**D**) Y27632 reduced the mRNA levels of cell cycle regulators *P21* and *P53* in P10 ASCs*.* No significant difference was observed in the mRNA level of *P16* gene. (**E**) After 72 h of cell seeding, the percentage of SA-β-Gal positive (blue) cells was decreased with Y27632 treatment in P10 ASCs. (**F**–**H**) The mRNA levels of osteogenesis-related genes (*Runx2, osteopontin,* and *osteocalcin*) were rejuvenated by Y27632 in P10 ASCs. (**I**) Y27632-treated P10 ASCs exhibited increased bone nodule formation in comparison to the control as indicated by the Alizarin Red staining area. *: *P* < 0.05

To further confirm whether the modulation of cell morphology plays a role in the rejuvenation of senescent ASCs mediated by OPN, we investigated whether altering cell shape by Y27632 could similarly induce rejuvenation in senescent ASCs. Y27632, as an inhibitor of Rho-associated kinases (ROCK), is known to modulate cell morphology and functionalities [30–32]. In contrast to the control group, Y27632-treated P10 ASCs exhibited a smaller cell area and an elongated cell shape (Fig. 8A, full-size image Fig. S8A), corresponding with the finding seen with P10 ASCs cultured on OPN-coating (Fig. 7). Furthermore, as the cell shape of senescent ASCs was modified by Y27632, we also studied how Y27632 affected senescence markers and osteogenic differentiation potential in P10 ASCs. Interestingly, Y27632 treatment resulted in the downregulation of gene expression related to cell cycle regulation levels (*P21* and *P53*) (Fig. 8B-D), as well as a reduction in the number of SA-β-Gal positive (blue) cells in P10 ASCs (Fig. 8E, full-size image Fig. S8B). Additionally, Y27632 treatment enhanced the mRNA expression levels of osteogenic genes, such as *Runx2, osteopontin,* and *osteocalcin*, in P10 ASCs (Fig. 8F-H), and improved the ability of P10 ASCs to form bone nodules, as indicated by Alizarin Red staining (Fig. 8I, full-size image Fig. S8C). Our study demonstrates that the regulation of cell shape is closely associated with the rejuvenation of cell senescence in ASCs and the restoration of their functional capabilities.

## Discussion

Emerging evidence suggests that senescent MSCs play a role in various age-related bone diseases and affects bone tissue regeneration and functions due to their compromised differentiation potential [33]. But recent evidence indicates that the senescent MSCs also show the age-related increase of the levels of secreted pro-inflammatory factors, known as inflammaging, resulting in a senescence drift to neighbouring cells [34], suggesting that investigating the paracrine effect of senescent stem cells, in addition to their differentiation potential, is also critical. OPN has shown promising effects for restoring impaired stem cell function [21, 22]. Nevertheless, its role in reversing stem cell senescence has not been thoroughly elucidated. Therefore, this study we investigated the effect of OPN on the senescence-related functions of ASCs. We developed an in vitro senescence model of ASCs using serial passaging for this purpose. Our findings revealed that OPN exert a rejuvenating effect on senescent ASCs, enhancing their osteogenic differentiation and trophic functions on osteoblasts. We further demonstrated that modulating cell morphology might play a role in OPN-mediated rejuvenation of senescent ASCs.

Cell senescence can be induced by various factors, resulting in several types of cell senescence, such as replicative senescence, oncogene-induced senescence, and oxidative stress-induced senescence [11]. Replicative senescence, one of the primary mechanisms in ageing-related diseases, is a phenomenon documented since the 1960s, where cells cease to proliferate while remaining metabolically active [12, 24]. Serial cell passaging is currently the primary method for in vitro MSCs expansion of MSCs[35]. However, at late passages, the extensive replication and expansion lead to an increased proportion of cells with growth arrest, known as senescent cells. As such, serial passaging is widely utilised in vitro to induce senescence, mimicking the process of replicative senescence observed in vivo [24, 35, 36]. It has been demonstrated that serial cell passaging can affect the characteristics of MSCs derived from different sources, such as bone marrow, adipose tissue, and umbilical cord [35–37]. In this study, we successfully induced a senescent phenotype in P10 adipose-derived stem cells (ASCs) by cell passaging, as evidenced by increased expression of senescence markers and a diminished osteogenic differentiation ability. These findings are in line with reports from other groups [35, 38–42] that have demonstrated an induced senescent phenotype and compromised differentiation ability in late-passaged stem cells. In addition, ASCs are increasingly appreciated for their trophic functions, as they secrete soluble factors such as chemokines, cytokines, and growth factors, which influence the immune system, cell senescence, and differentiation in the surrounding environment through a paracrine manner [43]. In the current study, we examined the trophic effects of ASCs on HOBs by culturing HOBs in conditioned media from either P4 or P10 ASCs. Our results revealed elevated gene expression levels of SASP factors in P10 ASCs, leading to a significant reduction in the trophic function of P10 ASCs and a compromised ability to induce osteogenesis in HOBs when compared to P4 ASCs. These results suggest that bone regenerative functions of ASCs, encompassing osteogenic differentiation and trophic activity, are impaired during senescence.

Considering the accumulations of bone regeneration-associated cells, including mesenchymal stem cells and other osteoprogenitor cells, in the ageing population [17], it is becoming critical to establish an anti-senescent niche for rejuvenating senescent cells and restoring their bone regenerative potential. This is particularly prevalent when seeking to improve bone healing ability or achieve large bone defect regeneration in the aged patients. Ideally, senescent cell rejuvenation strategies should target ameliorating detrimental effects while maintaining beneficial functions [44]. Current approaches for combatting senescence can be broadly categorized into pharmaceutical and genetic strategies. Pharmaceutical therapies, such as senomorphics and senolytics, play a crucial role in reversing cell senescence, as previously reported [44, 45]. Senomorphics focus on preventing the release of SASP factors, while senolytics rejuvenate senescent cells by eliminating the cells that release SASP factors [42]. However, it is essential to acknowledge the potential collateral damage to healthy cells in the local microenvironment when using senomorphics and senolytics. Alternatively, fully or partially reprogramming of ASCs through genetic modification enable the rejuvenation of senescent cells [9]. Several studies have investigated the changes in epigenetic landscapes of senescent stem cells. As previously noted, modulation of epigenetics such as DNA methylation and histone acetylation could significantly inhibit senescent phenotypes in ASCs [9, 46, 47]. Nonetheless, interfering directly with senescence-associated signalling pathways can pose risks to normal physiological processes [9]. Given the limitations of existing rejuvenation approaches, it is crucial to explore potential alternatives for targeting cell senescence. Our previous study demonstrated that Baghdadite bioceramics, could rejuvenate cell senescence in human osteoblasts by creating an anti-senescent local microenvironment to mitigate senescence-induced phenotypes. This highlights the potential of biomaterial-based strategies in rejuvenating cell senescence [17].

In this study, we hypothesised that the surface modification of biomaterials might shed new insights into rejuvenating senescent phenotype and regenerative function in ASCs. By coating OPN, a key non-collagenous component of bone extracellular matrix, on tissue culture plastic, we showed that P10 ASCs cultured on OPN-coated surfaces exhibited alleviated senescent phenotype and restored osteogenic differentiation abilities, compared to P10 ASCs on tissue culture plastic. In addition, we demonstrated a significant improvement in bone nodule formation by P10 ASCs on OPN-coated surfaces, further confirming the rejuvenation of osteogenesis by OPN. In addition, the gene expression levels of SASP factors in P10 ASCs were upregulated, compared to P4 ASCs cultured on tissue culture plastic, resulting in diminished trophic functions on HOBs in a paracrine manner. However, when HOBs were cultured in conditioned media collected from P10 ASCs on OPN-coated surfaces or on tissue culture plastic, OPN-coating downregulated SASP-related gene expressions in P10 ASCs, subsequently enhancing trophic function on HOBs. The positive influence was also observed in the osteogenesis of HOBs in OPN-P10 ASCs conditioned medium, suggesting that OPN has the potential to revive the trophic function of senescent ASCs. It is reported that the secreted factors from ASCs could be used in regenerative therapies [48]. A recent study demonstrated that paracrine molecules in exosome vesicles, secreted from young antler stem cells, could exert a trophic function on other cells by alleviating the senescent phenotype and enhancing osteogenic differentiation potentials [49]. However, molecules secreted from senescent ASCs, known as SASP, creates a pro-inflammatory microenvironment that compromises cell functions in their niche [48]. In our results, the trophic function of OPN-P10 ASCs on HOBs was enhanced compared to P10 ASCs, suggesting a rejuvenated trophic function of OPN-P10 ASCs. Given the observed rejuvenating effects of OPN on senescent ASCs, it is crucial to investigate the underlying mechanisms. Such study can provide insights for designing strategies to enhance the capacity for bone tissue regeneration in elderly patients.

One of the hallmarks of cell senescence is the morphological changes that occurs in senescent stem cells, including an enlarged cell area and flattened cell shape. Although the molecular mechanisms underlying these morphological changes during senescence remain to be explored, it has been revealed that stem cell mechanical properties play a pivotal role in mediating cell functions. This is achieved through cell surface receptors in response to various mechanical stimuli [28, 50]. This highlights the potential of modulating stem cell morphology to rejuvenate the function of senescent stem cells. An essential property of OPN is its ability to bind various cell-surface receptors, in particular integrins, and supports cell adhesion through its Arg-Gly-Asp (RGD) integrin recognition motif [51]. Integrins and their associated downstream signalling pathways have been increasingly appreciated for their roles in regulating cell shape and, subsequently, cellular senescence [52–54]. For instance, it has been reported that integrin β_3_ and its downstream signalling pathway possess regulatory effects on cellular senescence [53]. Furthermore, integrins can regulate endothelial cell shape by controlling the cytoskeleton [54], which consists of microfilaments, microtubules, and intermediate filaments, and is considered a critical player in the regulation of cell shape in age-related pathogenesis [28]. A previous study demonstrated that OPN could regulate cell morphology by interacting with α_ν_β_3_ integrin to activate the downstream P13K/uPA pathway and thus reorganising microtubules [55]. Additionally, existing evidence has shown that integrins act as a regulator of F-actin microfilament reorganisation, thereby regulating the cytoskeleton and cell shape [54]. Since the deficiency of F-actin microfilaments could be restored by the addition of OPN [51], OPN might modulate cell shape through the remodelling of microfilaments. In our study, we delved into alterations in cell area and shape with or without OPN to investigate whether the rejuvenating effects of OPN on ASC senescence are linked to the modulation of senescent cell morphology. Not surprisingly, our results demonstrated that the morphological alterations associated with cell senescence, as evidenced by the increased cell area and width-length (W/L) ratio in senescent P10 ASCs, were consistent with findings from previous studies [56–58]. A vital finding was that P10 ASCs cultured on OPN-coating displayed a smaller cell area and W/L ratio, compared to P10 ASCs on tissue culture plastic. We, therefore, postulated that OPN might modulate the senescent phenotype of ASCs through an integrin-cytoskeleton-cell shape axle.

To further elucidate the underlying mechanisms of morphology-associated rejuvenation in ASC senescence, we investigated the influences of Y27632 on modulating the cell shape of P10 ASCs. We measured the W/L ratio of P10 ASCs before and after Y27632 treatment and observed a substantial reduction in W/L ratio. This reduction suggests that Y27632, acting as a ROCK inhibitor, rejuvenates cell shape by disrupting the cytoskeleton and enhancing cell contraction in P10 ASCs. Y27632 is known to exert regulatory effects on cell shape, proliferation, and differentiation in stem cells by modulating the arrangement of the actin cytoskeleton [30–32]. Indeed, the changes in cell shape induced by Y27632 mirrored those observed with OPN-coating, effectively rejuvenating the senescent phenotype, and restoring the functionalities of senescent P10 ASCs. The alignment with prior research findings [30, 32] leads us to conclude that the rejuvenating effects of OPN on senescent ASCs may be linked to the observed alterations in cell shape and area. This suggests the potential application of modulating cell morphology as a strategy for rejuvenating cell senescence.

Although this study has demonstrated significant effects of OPN on ASC senescence in vitro, it has some limitations, and further studies are required for the future application of OPN in bone tissue engineering. First, as mentioned in previous sections, MSCs can be derived from various tissues such as bone marrow, adipose tissue, and umbilical cord. MSCs from different sources exhibit distinct characteristics, such as cell surface markers and lineage differentiation potentials [35–37]. While this study focused solely on stem cells generated from adipose tissue, exploring the effects of OPN on replicative senescence in MSCs from other sources would be interesting. Second, the rejuvenating effects of OPN on senescent ASCs need to be validated in relevant in vivo studies. Currently, we are in the process of incorporating OPN into appropriate scaffolds for a bone regeneration study in aged animals. Third, gaining a deeper understanding of the underlying mechanisms by which OPN mediates the anti-senescence effect of OPN on ASCs is necessary. In particular, investigating the roles of integrin-cytoskeleton-cell shape axle in regulating cell senescence would be of great interest.

## Conclusion

In summary, we developed an in vitro model of cell senescence in ASCs through serial passaging. In late passaged P10 ASCs, we successfully induced a senescent phenotype characterised by elevated expression levels of cell cycle regulator genes and a higher number of SA- β-Gal positive cells. Additionally, both the osteogenic differentiation ability of ASCs and their trophic function on HOBs were diminished due to serial cell passaging. Leveraging this ASC senescence model, we examined the effects of OPN on cell senescence and functionalities of ASC. Our findings demonstrate that OPN not only restored the senescent phenotype in P10 ASCs, but also restored their capacities of osteogenic differentiation and trophic function on HOBs. Furthermore, we evaluated the morphological changes in ASCs during the senescence process and revealed that OPN could modulate the cell shape and area of senescent ASCs, thereby rejuvenating both their phenotype and functions.

Overall, our research provides valuable insights into an anti-senescence strategy using OPN for rejuvenating senescent ASCs. This approach shows potential for integrating OPN into bone scaffold to confer anti-senescence properties. Moreover, the identified associations between cell senescence rejuvenation and morphology modulation offers potential guidance for future development of anti-senescence biomaterials, particularly for bone tissue regeneration applications in the elderly.

### Supplementary Information

Below is the link to the electronic supplementary material.

Supplementary file1 (DOCX 2859 KB)

## Data Availability

The data used to support the findings of this study are available from the corresponding author upon request.

## Declarations

The manuscript is the result of original research. Each author has participated in some parts of the conception, study design, and interpretation of the data. Furthermore, each author has been involved in the drafting of the article and concurred with the final version submitted. The material within has not been reported elsewhere except as abstract presentations. There are no interest conflicts to declare.
