# Supplementary material for: Osteopontin Rejuvenates Senescent Adipose-Derived Stem Cells and Restores their Bone Tissue Regenerative Function
Source: Stem Cell Rev Rep. 2024 Mar 12;20(4):1106–20. doi: 10.1007/s12015-024-10707-5 (PMC11087332; doi:10.1007/s12015-024-10707-5)
Supplement: Supplementary file 1 — Supplementary file1 (DOCX 2859 KB) [file 12015_2024_10707_MOESM1_ESM.docx]

**Supplementary Information for**

**Osteopontin rejuvenates senescent adipose-derived stem cells and restores their bone tissue regenerative function**

**Yiran Zhang^1^, Junni Zhang^1^, Pooria Lesani^1^, Zufu Lu^1*^, Hala Zreiqat^1*^**

^1^ Tissue Engineering & Biomaterials Research Unit, School of Biomedical Engineering, Faculty of Engineering and IT, The University of Sydney, NSW 2006, Australia

**^*^ Correspondence:**

Corresponding to: Hala Zreiqat; Zufu Lu

Email: [hala.zreiqat@sydney.edu.au](mailto:hala.zreiqat@sydney.edu.au); [zufu.lu@sydney.edu.au](mailto:zufu.lu@sydney.edu.au)

**Supplementary Full-Size Images**

**
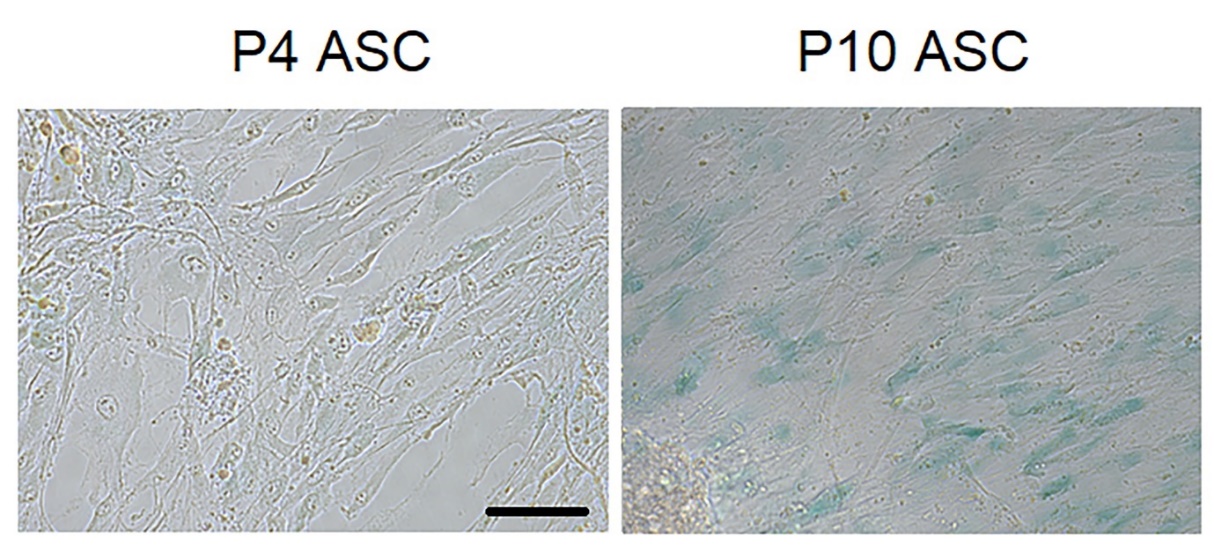
**

**Fig. S1 SA-β-Gal staining for P4 and P10 ASCs.** After 72 hours of cell culture, the observed percentage of SA-β-Gal positive staining cells was higher in P10 ASCs than that in P4 ASCs.


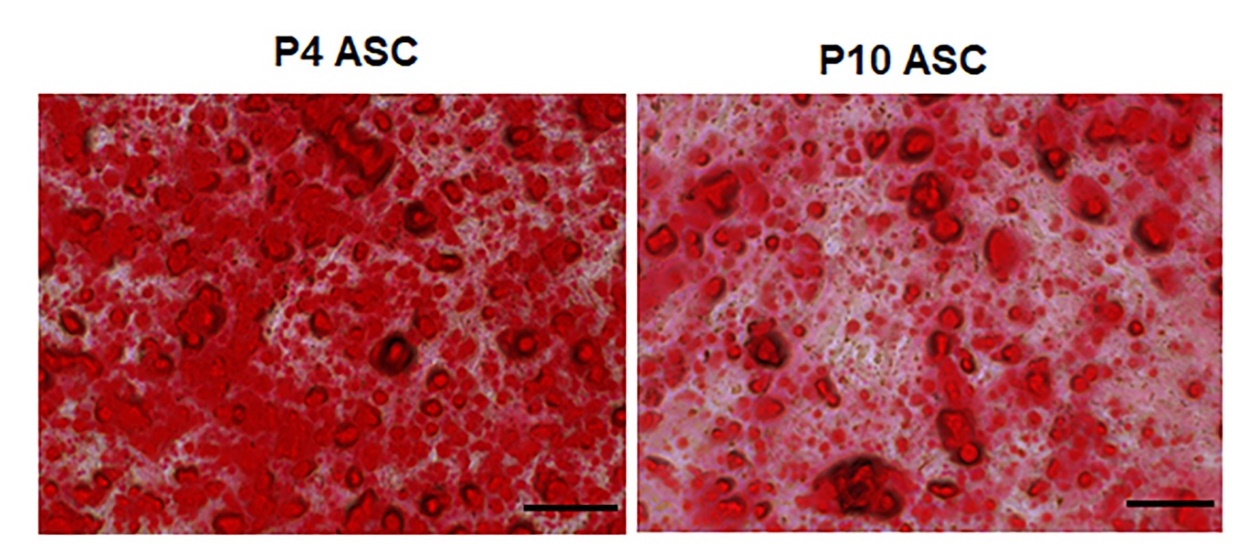


**Fig. S2** **Alizarin Red staining for P4 and P10 ASCs.** After being cultured in osteogenic media for 21 days, the Alizarin Red staining illustrated a significant decrease in mineralisation.


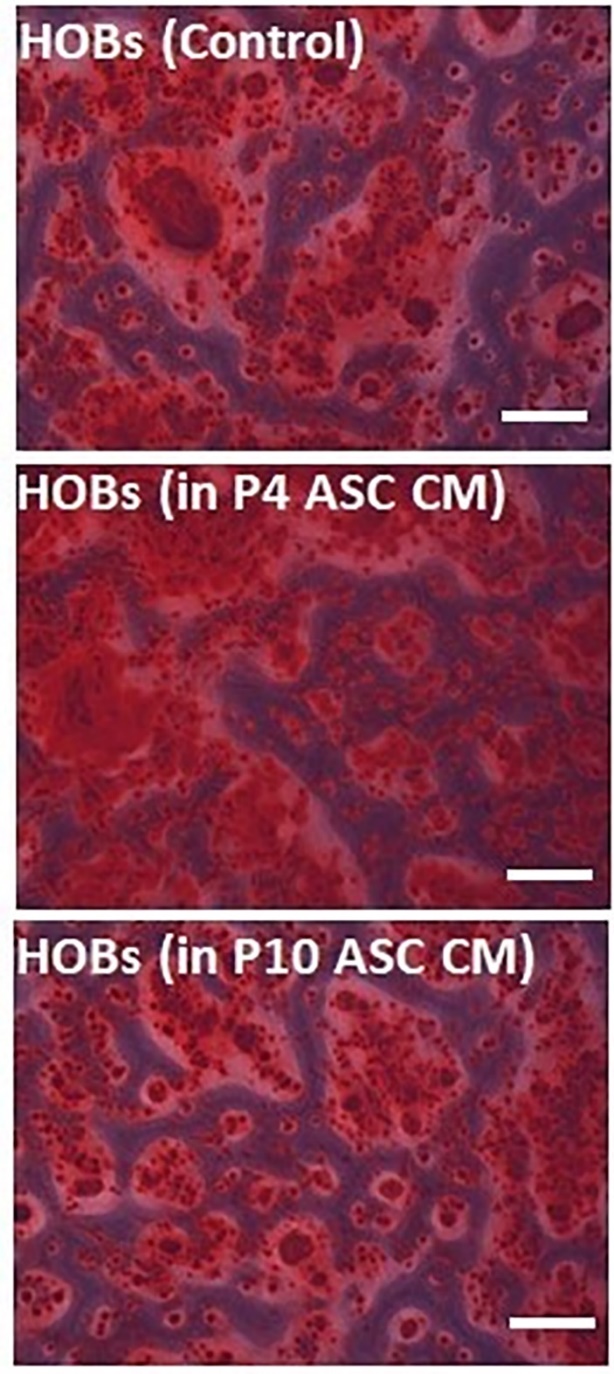


**Fig. S3 Alizarin Red staining of HOBs cultured in different conditioned media (CM).** The Alizarin Red staining area of HOBs cultured in P10 ASCs CM is significantly smaller than those cultured in P4 ASCs CM.


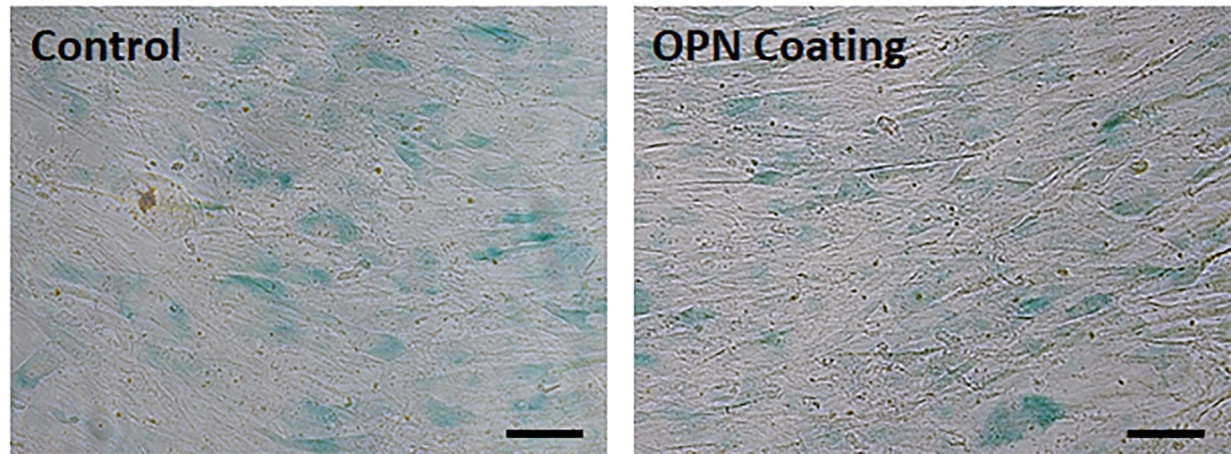


**Fig. S4** **SA-β-Gal staining for P10 ASCs cultured on tissue culture plastic and OPN coating.** After 72 hours of cell seeding, the percentage of SA-β-Gal positive (blue) cells was reduced in the OPN group in comparison to that in the control.


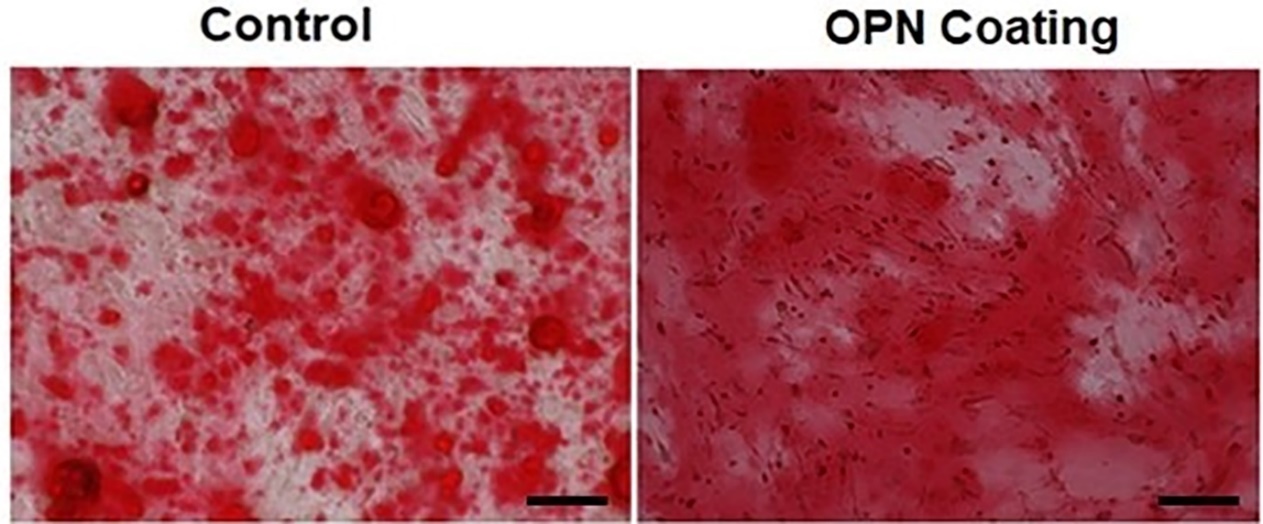


**Fig. S5** **Alizarin Red staining for P10 ASCs cultured on tissue culture plastic and OPN coating.** After culturing of 21 days, the Alizarin Red staining area in OPN group was increased compared to the P10 ASCs in the control.


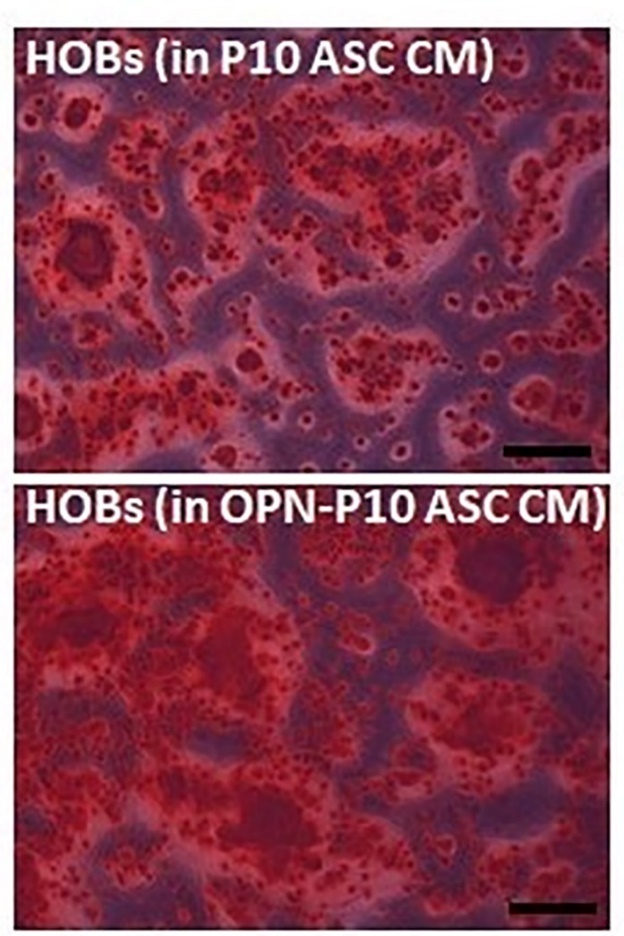


**Fig. S6** **Alizarin Red staining of HOBs cultured in different conditioned media (CM).** The Alizarin Red staining area of HOBs cultured in OPN-P10 ASCs CM implied an increased bone nodule formation than those cultured in P10 ASCs CM.


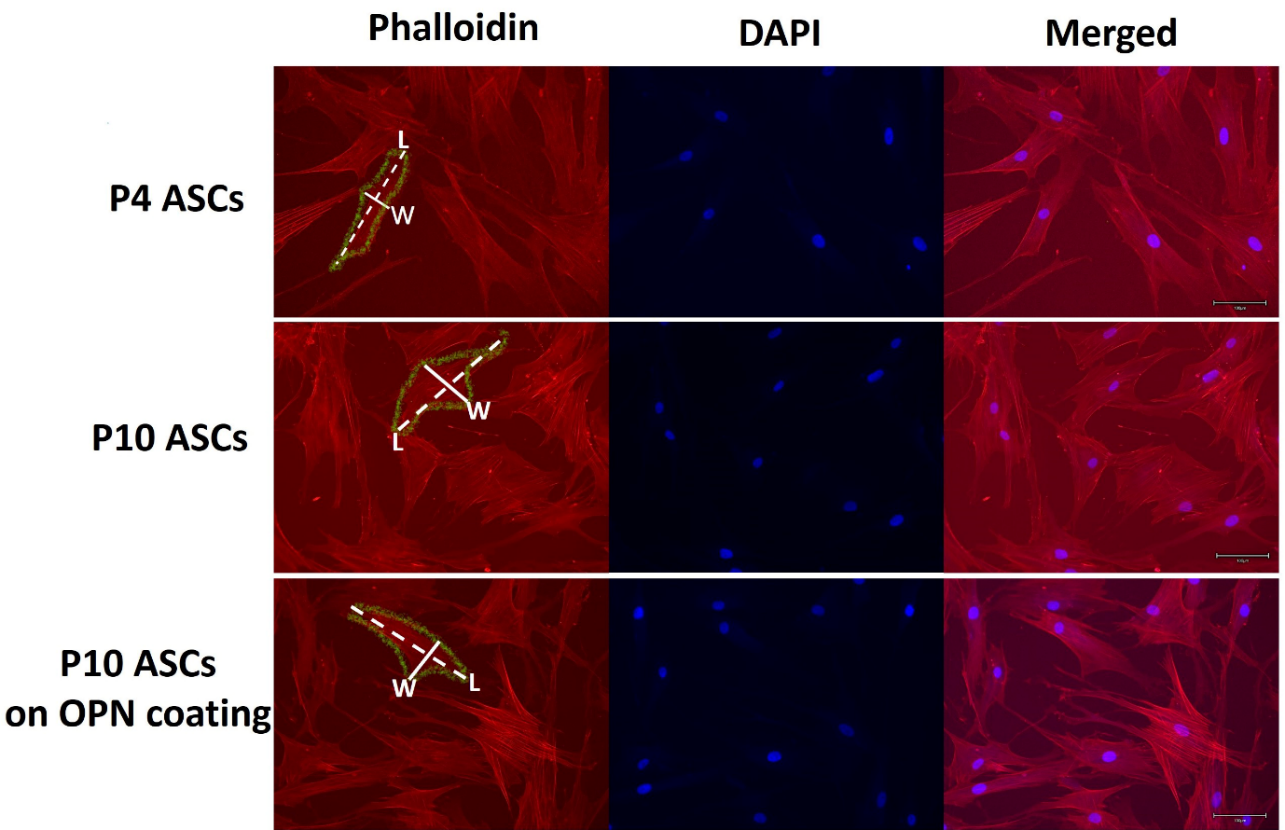


**Fig. S7 Immunofluorescence staining of P4 ASCs, P10 ASCs, and P10 ASCs on OPN-coating.** Phalloidin was used to stain the cytoskeleton of ASCs. The nuclei were stained blue with DAPI. The representative measurements of cell width (W) and length (L) were indicated by the solid line and the dashed line, respectively.


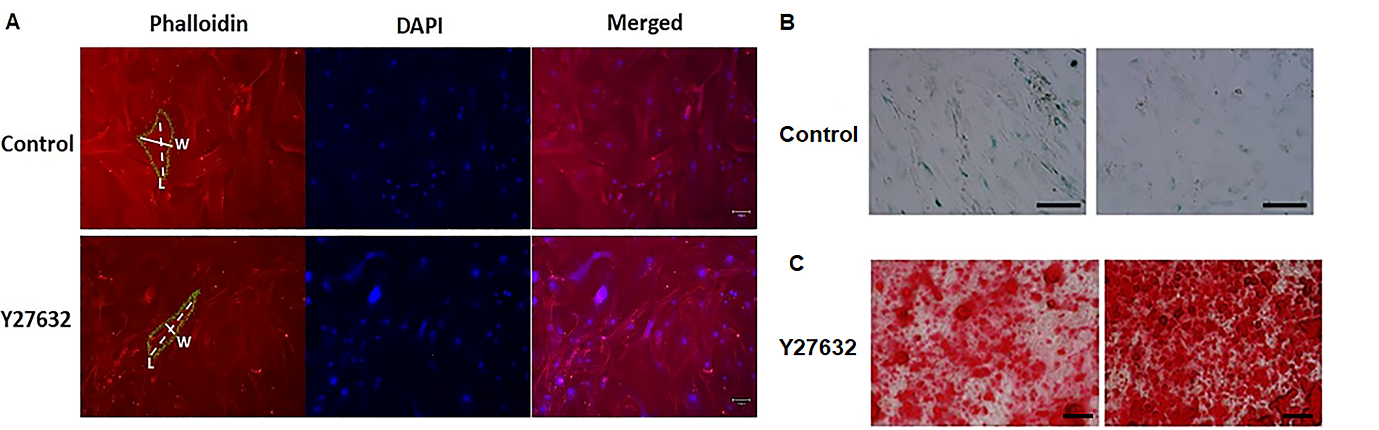


**Fig. S8** **The effects of Y27632 treatment on P10 ASCs**. (A) Phalloidin was used to stain the cytoskeleton of ASCs. The nuclei were stained blue with DAPI. The representative measurements of cell width (W) and length (L) were indicated by the solid line and the dashed line, respectively. The cell area and W/L ratio of Y27632-treated P10 ASCs were significantly reduced in contrast to the control. (B) After 72 hours of cell seeding, the percentage of SA-β-Gal positive (blue) cells was decreased with Y27632 treatment in P10 ASCs. (C) Y27632-treated P10 ASCs exhibited increased bone nodule formation in comparison to the control as indicated by the Alizarin Red staining area.
